# TNF-α Producing Innate Lymphoid Cells (ILCs) Are Increased in Active Celiac Disease and Contribute to Promote Intestinal Atrophy in Mice

**DOI:** 10.1371/journal.pone.0126291

**Published:** 2015-05-07

**Authors:** Irene Marafini, Ivan Monteleone, Davide Di Fusco, Maria Laura Cupi, Omero Alessandro Paoluzi, Alfredo Colantoni, Angela Ortenzi, Roberta Izzo, Simone Vita, Elisabetta De Luca, Giuseppe Sica, Francesco Pallone, Giovanni Monteleone

**Affiliations:** 1 Department of Systems Medicine, University of Rome “Tor Vergata”, Rome, Italy; 2 Department of Surgery, San Pietro Fatebenefratelli hospital, Rome, Italy; 3 Department of Surgery, University of Rome “Tor Vergata”, Rome, Italy; Harvard Medical School, UNITED STATES

## Abstract

Innate lymphoid cells (ILCs) are an emerging family of innate hematopoietic cells producing inflammatory cytokines and involved in the pathogenesis of several immune-mediated diseases. The aim of this study was to characterize the tissue distribution of ILCs in celiac disease (CD), a gluten-driven enteropathy, and analyze their role in gut tissue damage. ILC subpopulations were analyzed in lamina propria mononuclear cells (LPMCs) isolated from duodenal biopsies of CD patients and healthy controls (CTR) and jejunal specimens of patients undergoing gastro-intestinal bypass by flow cytometry. Cytokines and Toll-like receptors (TLR) were assessed in ILCs either freshly isolated or following incubation of control LPMC with peptidoglycan, poly I:C, or CpG, the agonists of TLR2, TLR3, or TLR9 respectively, by flow cytometry. The role of ILCs in gut tissue damage was evaluated in a mouse model of poly I:C-driven small intestine atrophy. Although the percentage of total ILCs did not differ between CD patients and CTR, ILCs producing TNF-α and IFN-γ were more abundant in CD mucosa compared to controls. ILCs expressed TLR2, TLR3 and TLR9 but neither TLR7 nor TLR4. Stimulation of LPMC with poly I:C but not PGN or CpG increased TNF-α and IFN-γ in ILCs. RAG1-deficient mice given poly I:C exhibited increased frequency of TNF-α but not IFN-γ/IL17A-producing ILCs in the gut and depletion of ILCs prevented the poly I:C-driven intestinal damage. Our data indicate that CD-related inflammation is marked by accumulation of ILCs producing TNF-α and IFN-γ in the mucosa. Moreover, ILCs express TLR3 and are functionally able to respond to poly I:C with increased synthesis of TNF-α thus contributing to small intestinal atrophy.

## Introduction

Celiac disease (CD) is a chronic enteropathy caused by dietary gluten in wheat, barley, and rye [1]. In genetically predisposed individuals, ingestion of gluten associates with activation of both innate and adaptive immune responses and production of elevated levels of inflammatory cytokines, with the downstream effect of causing villous atrophy and crypt hyperplasia [1, 2]. Several HLA-DQ2- and HLA-DQ8-restricted gluten peptides can promote the synthesis of interferon (IFN)-γ and interleukin (IL)-21 by CD4+ T cells [3, 4], while peptides p31-43 or p31-49 from α-gliadin stimulate innate immune cells (e.g. macrophages) to produce IL-15, a cytokine involved in the activation of cytotoxic cells [i.e. natural killer (NK) cells and CD8+ T cells]. [5] Additional cytokines over-produced in CD mucosa and supposed to amplify the tissue-damaging immune response include IL-17A, which is synthesised by gluten-non reactive CD4+ T cells and CD8+ T cells [6, 7] and TNF-α which is mainly produced by intraepithelial lymphocytes [8].

Recent studies have shown that wheat contains α-amilase/trypsin inhibitor (ATI) family members, a class of non-gluten proteins that interact with TLR4 and stimulate macrophages and dendritic cells to produce inflammatory cytokines [9]. Therefore, it is conceivable that CD-related inflammation is fuelled by additional factors other than gluten peptides.

Innate lymphoid cells (ILCs) are a family of hematopoietic cells involved in host defence against invading pathogens, immune homeostasis and tissue remodelling [10]. ILCs have lymphoid morphology, need IL-7 for their survival, and differentiate into cytokine-producing cells depending on specific genetic and environmental factors [11, 12]. ILCs share a requirement for the transcriptional repressor ID2 that inhibits the activity of the E protein transcription factors and is likely to antagonize B and T cell fates during ILC development [12]. Various subsets of ILCs have been characterized and like effector T cells, these cells express specific transcription factors, which are required to produce different types of cytokines [13]. Specifically, ILCs type 1 express the transcription factor T-bet and secrete the T helper type-1 (Th1)-related cytokine IFN-γ, ILCs type 2 express GATA3 and the chemokine receptor homologous molecule (CRTH2) and produce Th2-related cytokines, and ILCs type 3 express the transcription factor ROR-γt and synthesize predominantly IL-17, IL-22 and TNF-α [14, 15]. Although ILCs are present at a relatively small number in the gut, circumstantial evidence indicates that these cells play a substantial role in orchestrating detrimental responses, given their ability to establish rapid cytokine production and contribute to the polarisation of the subsequent adaptive immune response. In line with this, it was shown that both ILCs type 1 and ILCs type 3 infiltrate the intestinal mucosa of patients with Crohn’s disease, a Th1/Th17-associated inflammatory bowel disease, and contribute to pathology in murine models of colitis [16–18]. Studies in mice that lack a functional adaptive immune response have also shown that ILCs-derived IFN-γ is a potent inducer of bacteria-driven acute and chronic colitis [19], thus confirming the pathogenic role of ILCs.

The capacity of ILCs to rapidly produce pro-inflammatory cytokines such as TNF-α, IFN-γ and IL-17A that have a fundamental role in sustaining CD-related inflammation prompted us to characterize the tissue distribution and cytokines’ production of these subpopulations in CD and ascertain whether these cells contribute to CD-related tissue damage.

## Materials and Methods

### Patients and Samples

Duodenal biopsies were taken from 19 patients with active CD (ACD) at the time of diagnosis (median age 33, range 16–53), 14 patients with inactive (ICD, median age 34, range 18–50) on a gluten-free diet and 17 normal controls (median age 50, range 87–30). All patients with ACD were on a gluten-containing diet, were positive for both IgA anti-endomysium (EMA) and IgA anti-tissue transglutaminase 2 (TG2) and had villous atrophy on histological examination. ICD patients were on a gluten-free diet for at least 2 years (median years from diagnosis: 5 years, range: 2–15 years), were EMA and anti-TG2 negative and none of them had villous atrophy on histological examination. Control group included duodenal biopsies of 12 patients who underwent upper endoscopy for gastrointestinal symptoms, had no macroscopic/microscopic alteration, and were EMA and anti-TG2 negative. Additional control samples were jejunal specimens of 5 patients undergoing gastro-intestinal bypass for obesity. Each patient who took part in the study gave written informed consent and the independent local Ethics Committee of the University hospital of Tor Vergata approved the study protocol.

### Murine model of small intestinal atrophy

All reagents were from Sigma-Aldrich (Milan, Italy) unless specified. Fifteen 8 week-old female Bl/6 recombinase-activating gene1 (RAG1)-deficient mice (Charles River Laboratories, Wilmington, MA, USA) were given intra-peritoneally polyinosinic:polycytidylic acid (poly I:C) (15μg/g) dissolved in phosphate buffered saline (PBS) or PBS only (controls) and sacrificed 3 hours later through cervical dislocation. Small intestine was harvested for histology and lamina propria mononuclear cells (LPMC) isolation. To evaluate the contribution of ILCs in this model, mice were treated with anti CD90.2 depleting antibody (1 mg/mouse; Biolegend, San Diego, CA) or control Rat IgG (1mg/mouse; Biolegend) 24 and 12 hours before poly I:C injection. All experiments on animals were approved by the local Institutional Animal Care and Use Committee of the University of Tor Vergata.

### Cell isolation and culture

Human LPMC were isolated as previously described with minor modifications [20]. Briefly, biopsies taken from controls, ICD patients and ACD patients and surgical specimens of normal subjects were freed of mucus and epithelial cells in sequential steps with dithiothreitol (DTT) and ethylenediminetetracetic acid (EDTA) and then digested with liberase-tm (0,2mg/ml; Roche, Mannheim, Germany) and DNase I (0,2 mg/ml; Roche). LPMC were resuspended (1x10^6^/ml) in RPMI-1640 supplemented with 10% fetal bovine serum, penicillin (100μg/ml), streptomycin (100μg/ml), and gentamycin (50μg/ml; Lonza, Milan, Italy). Cells were either freshly stained for flow cytometry analysis or cultured with peptidoglycan (PGN, 10μg/ml), poly: IC (5μg/ml), CpG (1μg/ml) for 48 hours and then analyzed by flow cytometry. In each experiment after 48 hours stimulation, cell viability was evaluated with Annexin V/7AAD staining using flow-cytometry. Phorbol myristate acetate (PMA, 10 ng/ml), ionomycin (1 mg/ml), and brefeldin A (10 mg/ml; eBioscience, San Diego, CA) were added to the cultures in the last 5 hours in order to evaluate cytokine production. Peripheral blood mononuclear cells (PBMC) were isolated from EDTA-stabilized peripheral blood samples of patients and controls by Ficoll gradients and analyzed for the frequency of ILCs by flow-cytometry. LPMC were also isolated from small intestinal samples taken from control and poly I:C-treated mice with or without ILCs depletion [20].

### Histology

Small intestinal sections were stained with hematoxilin & eosin and morphological changes of villous architecture were evaluated using light microscopy (Olympus BX51, Byosystem82, Rome, Italy).

### Flow cytometry

Cells were immunostained with the following monoclonal anti-human antibodies: FITC anti-Lin3, V450 anti-CD56, APC-H7 anti-CD45, APC anti-IFN-γ, PE anti-TNF-α, PE Annexin V, 7AAD (all from Becton Dickinson, Milan, Italy), PercpCy5.5-CD127, PE anti-TLR3, PE anti-TLR9, APC anti-TLR4, PE anti-TLR2, APC anti-ROR-γt, PE anti-ROR-γt, PECy7 anti-T-bet, PE anti-IL-17A, AlexaFluor647 anti-IL-17A (all from eBioscience), PE anti-CRTH2 (Biolegend, San Diego, CA) and PE anti-TLR7 (R&D Systems). Cells were immunostained with the following anti-mouse antibodies: FITC antiCD90.2, hematopoietic lineage cocktail efluor 450, PECy7 anti-T-bet, APC anti-ROR-γt, PE anti-TNF-αPE anti-IL-17A (all from eBioscience), APC-Cy7 anti-CD45, PE anti-ROR-γt, PECy7 anti-IFN-γ (all from Becton Dickinson). In all experiments, appropriate isotype control IgGs (Becton Dickinson and eBioscience) and fluorescence minus one controls were used. All antibodies were used at 1:100 final dilution. For intracellular immunostaining, cells were fixed and permeabilized using staining buffer set and permeabilization buffer (both from eBioscience) according to the manufacturer’s instruction. Cells were analyzed by flow cytometry (FACSverse, BD Bioscience, San Jose, CA).

### Statistical analysis

Nonparametric methods were used for statistical analysis of the data using the Graph Pad Prism software version 5.00 (Graph Pad software Inc, La Jolla, CA, USA). Differences in the fractions of cytokines- and TLR-expressing ILCs in samples taken from controls, ICD and ACD patients were evaluated using the Mann–Whitney U-test. The Wilcoxon matched pairs test was used to compare groups in the *in vitro* study in which LPMC were stimulated with Poly I:C, while Mann-Whitney U-test was used to compare the fractions of cytokines-expressing ILCs following in vivo treatment of mice with Poly I:C.

## Results

### ILCs producing pro-inflammatory cytokines are significantly increased in the inflamed mucosa of active celiac disease patients

**Fig 1:**
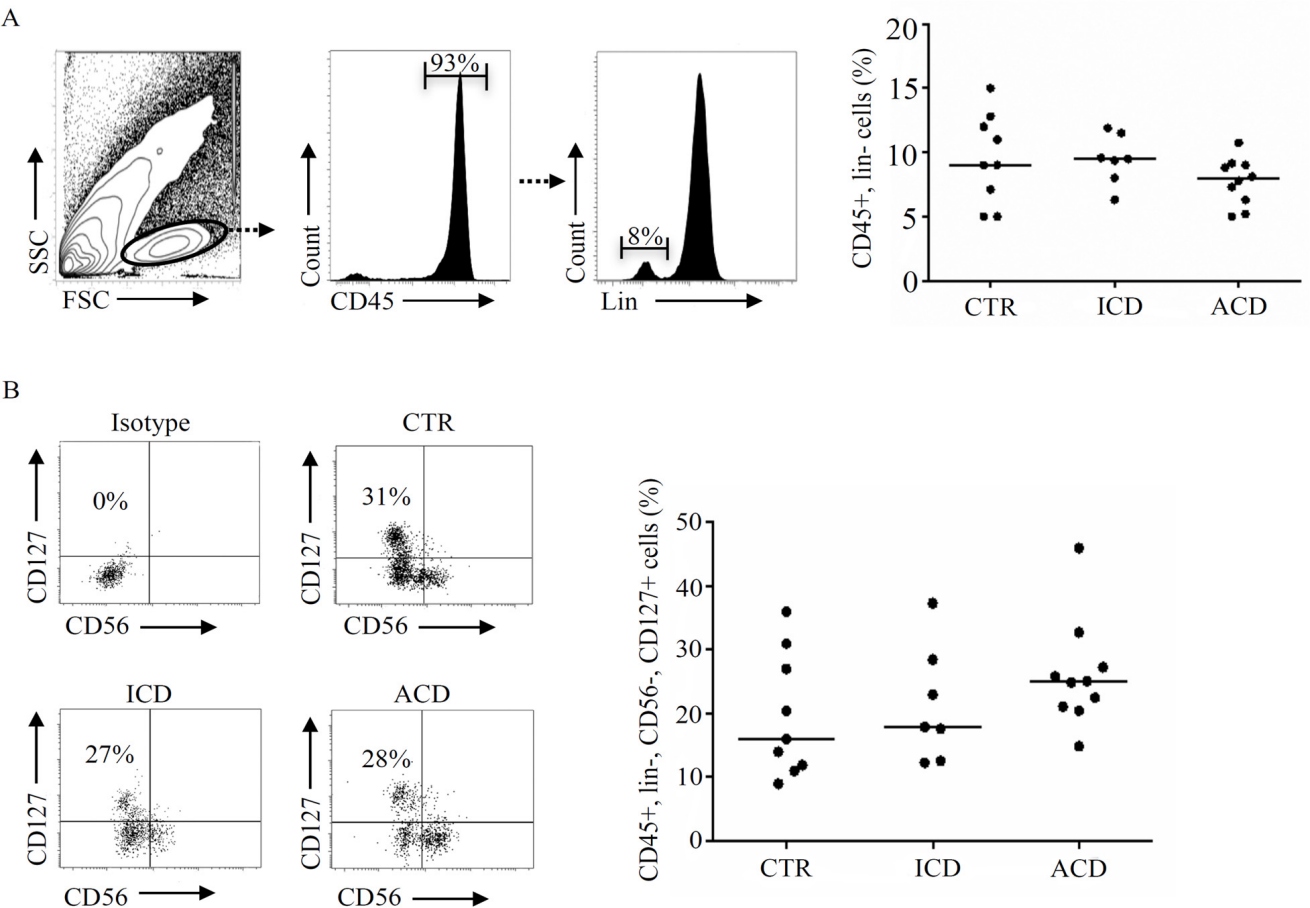
The percentages of CD45+, lineage (lin)- cells and CD45+, lin-, CD56-, CD127+ ILCs in active celiac disease (ACD) did not differ from those in normal controls (CTR) and inactive celiac disease (ICD). A. Lamina propria mononuclear cells (LPMC), isolated from 9 CTR, 7 ICD patients and 10 ACD patients, were gated on the lymphocytic area in the FSC/SSC plot, then on the CD45+ population and next on the lin- population. Right panel: percentages of CD45+, lin- cells in CTR, ICD and ACD. Each point in the graph indicates the percentage of positive cells in a single sample of a single patient. The horizontal bars represent the median values. B. CD45+, lin- LPMC were stained with CD127 and CD56 antibodies. Left panels: representative dot-plots showing the percentages of CD56-, CD127+ ILCs. Right panel shows the percentages of CD45+, lin-, CD56-, CD127+ ILCs in CTR, ICD patients and ACD patients. Each point in the graph indicates the percentage of positive cells in a single sample of a single patient. The horizontal bars represent the median values.

ILCs express CD45 and CD127 (IL-7 receptor α) and are negative for classical markers of hematopoietic cells. Initially, we analyzed the distribution of CD45-expressing cells negative for CD3, CD14, CD19 and CD20 (lineage negative) in the mucosa of patients with ACD, patients with ICD and controls. To this end, LPMC were immunostained with CD45 and a cocktail of antibodies (lineage cocktail) for CD3, CD19, CD20 and CD14 and then analyzed by flow cytometry (Fig 1A). The percentage of CD45-positive, lineage negative (lin-) cells in ACD did not differ from that in ICD and controls (Fig 1A, right panel). Next, by gating on CD45+ cells that were lin-, lacked CD56 and expressed CD127, we detected ILCs. The fractions of ILCs did not differ among the 3 groups (Fig 1B). ILCs were barely detectable in PBMC samples of patients and controls (not shown).

**Fig 2:**
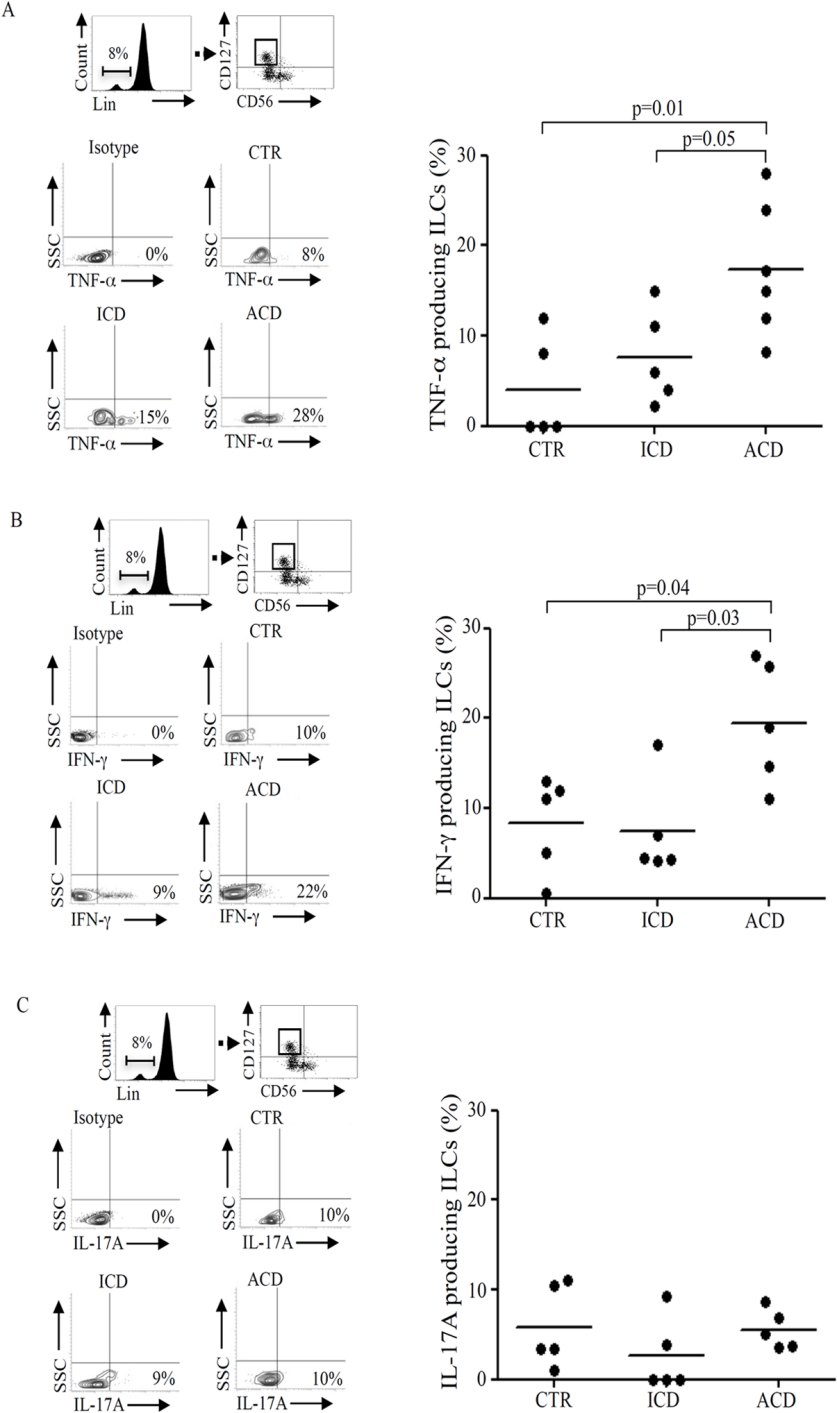
Innate lymphoid cells (ILCs) producing TNF-α and IFN-γ are significantly increased in the inflamed mucosa of celiac disease patients. A. Lamina propria mononuclear cells (LPMC), isolated from 5 normal controls (CTR), 5 inactive celiac disease (ICD) patients and 6 active celiac disease (ACD) patients, were gated on CD45+/lin- population and stained with CD56, CD127, and TNF-α. Representative dot-plots showing TNF-α expression in CD45+, Lin-, CD56-, CD127+ ILCs in CTR, ICD and ACD. Right panel shows the percentages of CD45+, lin-, CD56-, CD127+ ILCs expressing TNF-α in CTR, ICD patients and ACD patients. Each point in the graph indicates the percentage of positive cells in a single sample of a single patient. The horizontal bars represent the median values. B. LPMC, isolated from 5 CTR, 5 ICD patients and 5 ACD patients, were gated on CD45+/lin- population and stained with CD56, CD127, and IFN-γ. Representative dot-plots show IFN-γ expression in CD45+, Lin-, CD56-, CD127+ ILCs in CTR, ICD and ACD. Right panel shows the percentages of CD45+, lin-, CD56-, CD127+ ILCs expressing IFN-γ in CTR, ICD patients and ACD patients. Each point in the graph indicates the percentage of positive cells in a single sample of a single patient. The horizontal bars represent the median values. C. LPMC, isolated from 5 CTR, 5 ICD patients and 5 ACD patients, were gated on CD45+/lin- population and stained with CD56, CD127, and IL-17A. Representative dot-plots show IL-17A expression in CD45+, Lin-, CD56-, CD127+ ILCs in CTR, ICD and ACD. Right panel shows the percentages of CD45+, lin-, CD56-, CD127+ ILCs expressing IL-17A in CTR, ICD patients and ACD patients. Each point in the graph indicates the percentage of positive cells in a single sample of a single patient. The horizontal bars represent the median values.

To ascertain whether ILCs could make a contribution to CD-associated cytokine response, we analyzed the production of TNF-α, IFN-γ and IL-17A in ILCs. In ACD patients both TNF-α and IFN-γ-producing ILCs were significantly increased compared to ICD patients and controls (Fig 2A and 2B), while the fraction of IL-17A-producing ILCs did not differ among groups (Fig 2C). Assessment of mean fluorescence intensity (MFI) showed no difference among groups in terms of TNF-α -producing ILCs, while MFI of IFN-γ -producing ILCs was significantly increased in ACD patients compared to ICD patients and controls. Moreover, MFI of IL-17A-producing ILCs was significantly decreased in ICD as compared with ACD and controls with no difference between these two groups. (S1A–S1C Fig)

Altogether these data indicate that, in ACD, ILCs producing pro-inflammatory cytokines, such TNF-α and IFN-γ are significantly increased in ACD.

### ILCs express toll like receptors (TLRs) and respond to TLR ligands

**Fig 3:**
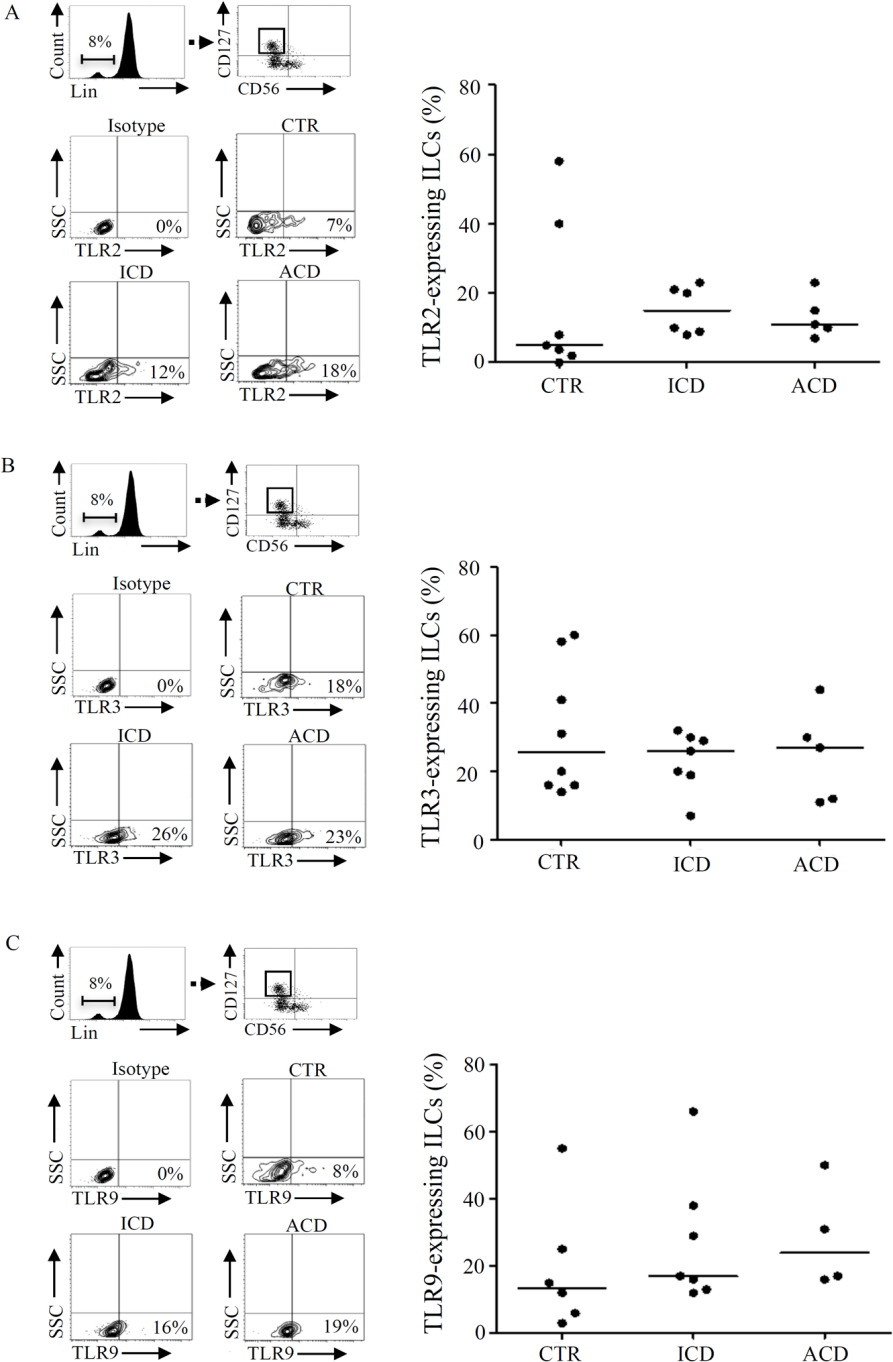
Innate lymphoid cells (ILCs) express toll like receptors (TLRs). A. Lamina propria mononuclear cells (LPMC), isolated from 7 controls (CTR), 6 inactive celiac disease (ICD) patients and 5 active celiac disease (ACD) patients, were gated on CD45+/lin- population and stained with CD56, CD127, and TLR2. Representative dot-plots show TLR2 expression in CD45+, lin-, CD56-, CD127+ cells in CTR, ICD and ACD. Right panel: percentages of CD45+, lin-, CD56-, CD127+ ILCs expressing TLR2 in CTR, ICD patients and ACD patients. Each point in the graph indicates the percentage of positive cells in a single sample of a single patient. The horizontal bars represent the median values. B. LPMC, isolated from 8 CTR, 7 ICD patients and 5 ACD patients, were gated on CD45+/lin- population and stained with CD56, CD127, and TLR3. Representative dot-plots show TLR3 expression in CD45+, lin-, CD56-, CD127+ cells in CTR, ICD and ACD. Right panel: percentages of CD45+, lin-, CD56-, CD127+ ILCs expressing TLR3 in CTR, ICD patients and ACD patients. Each point in the graph indicates the percentage of positive cells in a single sample of a single patient. The horizontal bars represent the median values. C. LPMC, isolated from 6 CTR, 7 ICD patients and 4 ACD patients, were gated on CD45+/lin- population and stained with CD56, CD127, and TLR9. Representative dot-plots show TLR9 expression in CD45+, lin-, CD56-, CD127+ cells in CTR, ICD and ACD. Right panel: percentages of CD45+, lin-, CD56-, CD127+ ILCs expressing TLR9 in CTR, ICD patients and ACD patients. Each point in the graph indicates the percentage of positive cells in a single sample of a single patient. The horizontal bars represent the median values.

Lymphoid tissue inducer-like cells, members of the ILCs family, express functional TLR2 and respond to TLR2 ligands with increased production of cytokines [21]. This observation well fits with the role of ILCs in innate immune response against pathogens [22, 23]. Therefore, we next examined whether, in the human duodenum, ILCs express TLRs. To this end, LPMC were immunostained with TLR 2, 3, 4, 7 and 9 and ILCs were then identified as indicated above. Approximately 10% of ILCs expressed TLR2, 26% expressed TLR3 and 20% expressed TLR9 with no significant difference among groups (Fig 3A–3C). In contrast, TLR4 and TLR7 were expressed by CD45+, lin+ LPMC but not ILCs (S2 Fig).

**Fig 4:**
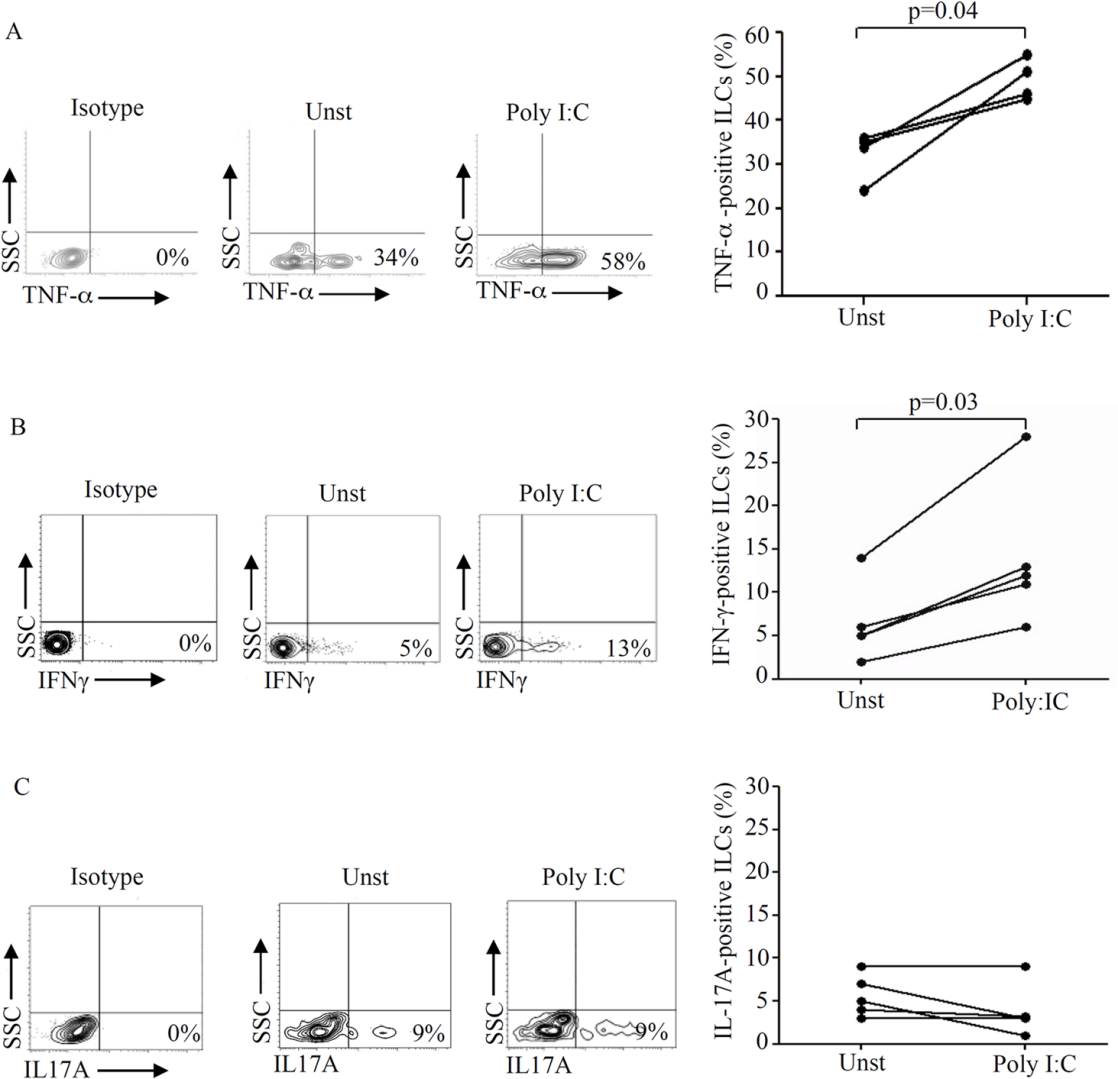
Stimulation of lamina propria mononuclear cells (LPMC) with poly I:C significantly increases the production of TNF-α and IFN-γ by innate lymphoid cells (ILCs). A. LPMC, isolated from jejunal specimens of 4 controls, were stimulated with poly I:C and analyzed by flow cytometry. LPMC were gated on CD45+/lin- population and stained with CD56, CD127, and TNF- α. Representative dot-plots show TNF-α expression in CD45+, lin-, CD56-, CD127+ ILCs in cultures of LPMC either left unstimulated (Unst) or stimulated with poly I:C. Right panel: percentages of CD45+, lin-, CD56-, CD127+ ILCs expressing TNF-α in unstimulated cells or after stimulation with poly I:C. Each point in the graph indicates the percentage of positive cells in a single sample of a single patient. Each line connects unstimulated and stimulated wells of a single patient. B. LPMC, isolated from jejunal specimens of 5 controls, were stimulated with poly I:C and analyzed by flow cytometry. LPMC were gated on CD45+/lin- population and stained with CD56, CD127, and IFN-γ. Representative dot-plots show IFN-γ expression in CD45+, lin-, CD56-, CD127+ ILCs in cultures of LPMC either left unstimulated (Unst) or stimulated with poly I:C. Right panel: percentages of CD45+, lin-, CD56-, CD127+ ILCs expressing IFN-γ in unstimulated cells or after stimulation with poly I:C. Each point in the graph indicates the percentage of positive cells in a single sample of a single patient. Each line connects unstimulated and stimulated wells of a single patient. C. LPMC, isolated from jejunal specimens of 5 controls, were stimulated with poly I:C and analyzed by flow cytometry. LPMC were gated on CD45+/lin- population and stained with CD56, CD127, and IL-17A. Representative dot-plots show IL-17A expression in CD45+, lin-, CD56-, CD127+ ILCs in cultures of LPMC either left unstimulated (Unst) or stimulated with poly I:C. Right panel: percentages of CD45+, lin-, CD56-, CD127+ ILCs expressing IL-17A in unstimulated cells or after stimulation with poly I:C. Each point in the graph indicates the percentage of positive cells in a single sample of a single patient. Each line connects unstimulated and stimulated wells of a single patient.

The number of LPMC yielded from pinch biopsy samples is not sufficient to carry out functional studies. Therefore, to evaluate whether TLR ligands regulate ILC-derived cytokine production, LPMC were isolated from jejunal specimens and cultured in the presence of PGN, poly: IC and CpG, the ligands of TLR2, TLR3 and TLR9, respectively. After 48 hours, LPMC were immunostained and production of cytokines by ILCs was evaluated by flow-cytometry. LPMC viability, as evaluated by flow-cytometry using Annexin V/7AAD staining, was more than 90% in all the experiments. Stimulation of LPMC with poly I:C, but neither with PGN nor CpG (not shown), significantly enhanced the production of TNF-α and IFN-γ by CD45+, lin-, CD127+, CD56- ILCs (Fig 4A and 4B). In contrast, no significant change in IL-17A production by ILCs was seen following stimulation of LPMC with poly I:C (Fig 4C) or other TLRs ligands (not shown).

### Depletion of innate lymphoid cells attenuates poly I:C-induced villous atrophy

**Fig 5:**
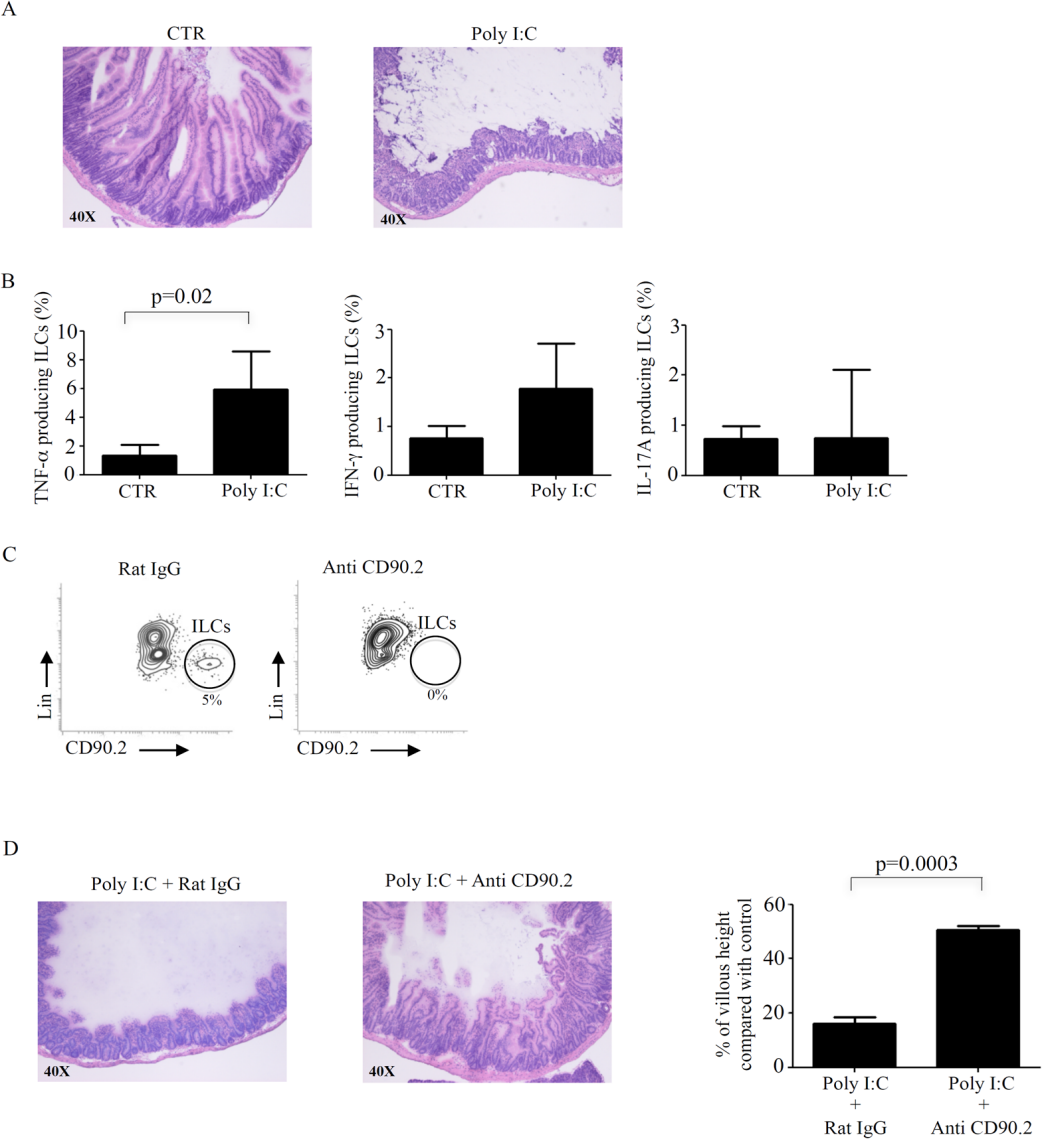
Depletion of innate lymphoid cells (ILCs) attenuates poly I:C-induced small intestinal atrophy. A. Hematoxilyn & eosin (HE)-stained frozen sections of representative small intestinal tissues taken from control RAG1 deficient (-/-) mice injected with phosphate buffered saline (CTR) and poly I:C-treated RAG1 (-/-) mice. B. Lamina propria mononuclear cells (LPMC) were isolated from the small intestine of control RAG1 (-/-) mice and poly I:C-treated RAG1 (-/-) mice, gated on CD45+, lineage (lin)-, CD90.2+ cells and stained with TNF-αIFN-γ and IL-17A. Graphs show the percentages of CD45+, lin- (CD3-, B220-, CD11b-, TER-119-, GR-1-), CD90.2+ ILCs expressing TNF-αIFN-γ and IL-17A in control RAG1 (-/-) mice (n = 3) and poly I:C-treated RAG1 (-/-) mice (n = 6). Data are indicated as mean and standard deviation of all experiments. C. Representative contour plots of Rag1 (-/-) mice treated with poly I:C after injection with control Rat IgG (n = 3) or anti-CD90.2 depleting antibody (n = 3). LPMC were gated on CD45+, lin-, CD90.2+ ILCs. D. HE-stained frozen sections of representative small intestinal tissues taken from RAG1 (-/-) mice treated with poly I:C after injection with control Rat IgG (n = 3) or anti-CD90.2 depleting antibody (n = 3). In the right panel is represented the percentage of villous height in RAG1 (-/-) mice treated with poly I:C after injection with control Rat IgG or anti-CD90.2 depleting antibody compared to control RAG1 (-/-) mice. Data are indicated as mean and standard deviation of all experiments.

In mice intra-peritoneal injection of poly I:C induces CD-like enteropathy, which is characterized by activation of immune cells and villous atrophy [24]. Since TNF-α has been involved in the intestinal damage in this model and human intestinal ILCs respond to poly I:C with enhanced production of TNF-α we explored the possibility that ILCs could play a role in the pathogenesis of poly I:C-driven villous atrophy. To this end, RAG1-deficient mice, which lack lymphocytes and represent an excellent system to study ILCs functions, were given poly I:C. Treatment of mice with poly I:C caused a marked flattening of small intestinal villi as compared to mice treated with PBS (Fig 5A). TNF-α producing ILCs were significantly increased in poly I:C-treated mice compared to control mice, while there was no difference in terms of IFN-γ and IL-17A-expressing ILCs (Fig 5B). To determine whether ILCs had a functional role in poly I:C-driven small intestinal atrophy, these cells were depleted using a specific anti-CD90.2 antibody [19], before poly I:C administration. As expected mice treated with anti-CD90.2 were devoid of CD45+, lin-, CD90.2 positive cells (Fig 5C). Depletion of ILCs significantly attenuated villous atrophy (Fig 5D).

## Discussion

In this study we determined the frequency and contribution of ILCs in the cytokine response in CD. Our data confirm and expand on previous studies showing that ILCs are a minority of hematopoietic cells in the human gut [16–18]. Although there is no difference in the percentages of ILCs between ACD patients and controls, analysis of ILCs subtypes reveals that TNF-α - and IFN-γ-producing ILCs are more frequent in ACD as compared to controls. Control group included both healthy, non-obese patients undergoing upper endoscopy and obese patients subjected to surgical gastrointestinal bypass. Since no difference was seen in terms of cytokines-producing ILCs and TLRs expression between these two groups, we considered them as a unique category of controls.

Cytokines play a decisive role in orchestrating the pathologic process leading to villous atrophy in CD. Major cell sources of inflammatory cytokines are adaptive and innate immune cells as well as non-immune cells [1, 2]. Results from the present studies indicate that ILCs represent a further source of effector cytokines in CD, thus confirming studies in other systems showing that ILCs can produce a complex repertoire of cytokines, that resembles that synthesized by polarized CD4+ T cells [10].

The factors/mechanisms underlying the accumulation of TNF-αand IFN-γ-producing ILCs in CD remain to be ascertained. It has been suggested that specific environmental cues can favour the generation of ILC subtypes as well as shifting from a specific subset to another one [25]. In this context, for example, it has been shown that commensal microbiota-driven IL-7 is a key determinant in the maintenance of ROR-γt in ILCs type 3 as well as aryl hydrocarbon receptor, a ligand-dependent transcriptional factor that binds to dietary and microbial metabolites, influences the function of ILCs type 3 in the gut [26]. Moreover, IL-23-responsive ILCs type 3 that produce IL-22 can be induced to synthesize IFN-γ when cultured either with Th1-inducing stimuli, such as IL-12, or in the presence of IL-7 and IL-2 and stimulated with IL-23 [16]. Since ILCs lack a T cell receptor, it is unlikely that differentiation of ILCs type 1 and ILCs type 3 occurs in an antigen-specific manner. In contrast, non-gluten proteins (e.g. ATI), contained in wheat and related cereals and signalling through TLR, can be involved in this process as these proteins activate innate immune cells and here we demonstrate that ILCs express TLRs and respond to TLR ligands with enhanced production of TNF-α and IFN-γ Another possibility is that cytokines produced in the early phase of the immune response to gluten make a valid contribution to the generation and/or expansion of TNF-α and IFN-γ-producing ILCs. Finally it is conceivable that the preferential accumulation of TNF-α and IFN-γ-producing ILCs in ACD is the result of the colonization of the gut mucosa with specific pathogens, in line with the demonstration that bacterial/viral infections enhance the risk of CD [27] and changes in the duodenal bacterial community have been described in CD patients [28].

Since we were not able to purify a sufficient number of ILCs to carry out the functional studies, at this stage we cannot exclude the possibility that the positive effect of poly I:C on ILC-derived TNF-α and IFN-γ production is indirect as a result of the ability of such stimuli to affect the function of additional cell types contained in the LPMC preparations.

The evidence that normal mice injected with TNF-α develop severe enteropathy [29] and the rapid response of ILCs to poly I:C administration with increased TNF-α production suggest a possible role for ILCs in the early phases of tissue damage in CD. This hypothesis is supported by the demonstration that depletion of ILCs in RAG1-deficient mice attenuates poly I:C-induced gut atrophy. Moreover these findings raise the possibility that viral products can contribute to the initiation and/or perpetuation of the detrimental immune response in CD in line with several epidemiological/experimental data supporting the involvement of viral infections in CD pathogenesis [27, 30].

The function of ILCs in immune-mediated pathologies is not yet fully understood but accumulating evidence suggests that cytokines produced by ILCs contribute to expand detrimental responses in various organs. For instance, ILCs type 2 producing IL-13 participate to disease pathology in experimental models of allergic asthma [31, 32] and virus-induced asthma exacerbation[33]. Similarly, ILCs type 1 and ILCs type 3 have been involved in the development and progression of various models of colitis in mice [16, 19], as well as IL-13-producing ILCs type 2 have been described in the oxazolone-induced model of colitis [10], which shows some immunological similarities with ulcerative colitis. In conclusion, this study is the first to provide a phenotypic characterization of ILCs in CD demonstrating that ILCs are a source of effector cytokines in this disorder and contribute to gut damage in a mouse model of poly I:C-driven small intestinal atrophy.

## Supporting Information

S1 Fig Mean fluorescence intensity (MFI) values of TNF-α-, IFN-γ-(B), and IL-17A-expressing (C) ILCs in controls (CTR), inactive celiac disease (ICD) and active celiac disease (ACD) patients. Each point in the graph indicates the MFI value in a single sample of a single patient. The horizontal bars represent the median values.
(TIF)

S2 Fig Toll like receptor (TLR) 4 and TLR7 are expressed by CD45+, lineage (lin) + cells but not innate lymphoid cells (ILCs). Representative dot-plot showing TLR4 and TLR7 in lamina propria mononuclear cells (LPMC) isolated from the duodenum of a normal control and stained for CD45, lin, TLR4 and TLR7 and analyzed by flow-cytometry. The example is representative of 10 experiments in which LPMC of 5 controls, 2 inactive celiac disease patients and 3 active celiac disease patients were analyzed. Similar results were obtained in all these experiments.
(TIF)
