# Supplementary figures and images for: TNF-α Producing Innate Lymphoid Cells (ILCs) Are Increased in Active Celiac Disease and Contribute to Promote Intestinal Atrophy in Mice
Source: PLoS One. 2015 May 7;10(5):e0126291. doi: 10.1371/journal.pone.0126291 (PMC4423916; doi:10.1371/journal.pone.0126291)

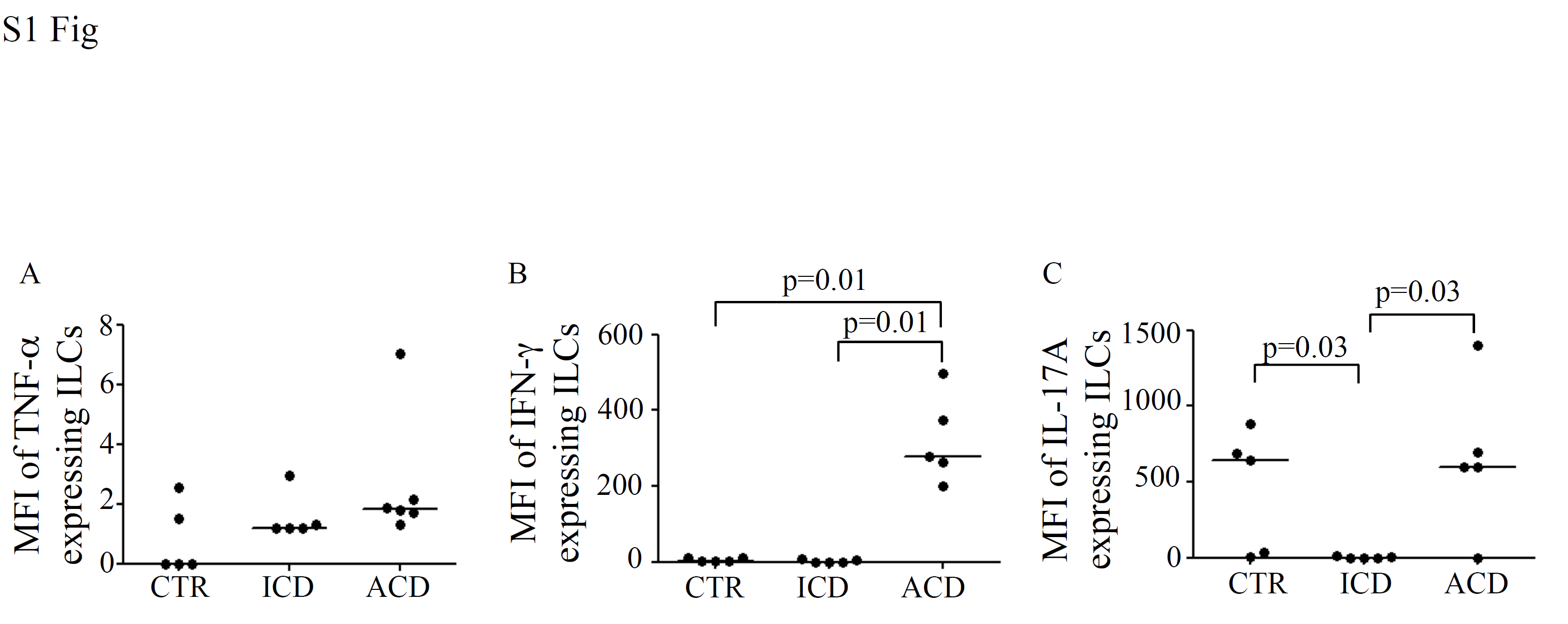

Supplement: S1 Fig — Each point in the graph indicates the MFI value in a single sample of a single patient. The horizontal bars represent the median values. (TIF) [file pone.0126291.s001.tif]

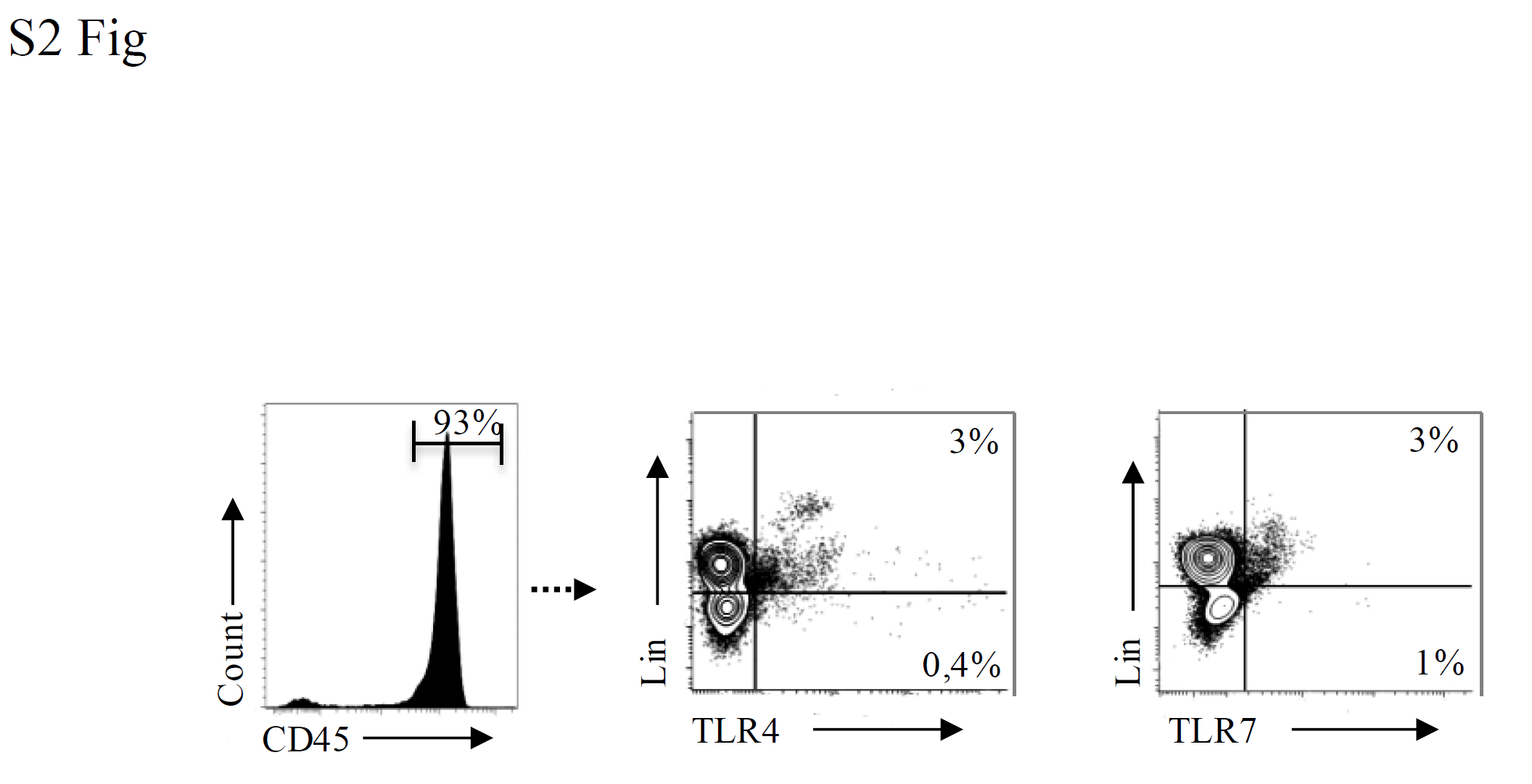

Supplement: S2 Fig — Representative dot-plot showing TLR4 and TLR7 in lamina propria mononuclear cells (LPMC) isolated from the duodenum of a normal control and stained for CD45, lin, TLR4 and TLR7 and analyzed by flow-cytometry. The example is representative of 10 experiments in which LPMC of 5 controls, 2 inactive celiac disease patients and 3 active celiac disease patients were analyzed. Similar results were obtained in all these experiments. (TIF) [file pone.0126291.s002.tif]
